# Identification of a novel 5-aminomethyl-2-thiouridine methyltransferase in tRNA modification

**DOI:** 10.1093/nar/gkad048

**Published:** 2023-02-10

**Authors:** Gyuhyeok Cho, Jangmin Lee, Jungwook Kim

**Affiliations:** Department of Chemistry, Gwangju Institute of Science and Technology, Gwangju 61005, Korea; Department of Chemistry, Gwangju Institute of Science and Technology, Gwangju 61005, Korea; Department of Chemistry, Gwangju Institute of Science and Technology, Gwangju 61005, Korea

## Abstract

The uridine at the 34th position of tRNA, which is able to base pair with the 3′-end codon on mRNA, is usually modified to influence many aspects of decoding properties during translation. Derivatives of 5-methyluridine (xm^5^U), which include methylaminomethyl (mnm-) or carboxymethylaminomethyl (cmnm-) groups at C5 of uracil base, are widely conserved at the 34th position of many prokaryotic tRNAs. In Gram-negative bacteria such as *Escherichia coli*, a bifunctional MnmC is involved in the last two reactions of the biosynthesis of mnm^5^(s^2^)U, in which the enzyme first converts cmnm^5^(s^2^)U to 5-aminomethyl-(2-thio)uridine (nm^5^(s^2^)U) and subsequently installs the methyl group to complete the formation of mnm^5^(s^2^)U. Although mnm^5^s^2^U has been identified in tRNAs of Gram-positive bacteria and plants as well, their genomes do not contain an *mnmC* ortholog and the gene(s) responsible for this modification is unknown. We discovered that MnmM, previously known as YtqB, is the methyltransferase that converts nm^5^s^2^U to mnm^5^s^2^U in *Bacillus subtilis* through comparative genomics, gene complementation experiments, and *in vitro* assays. Furthermore, we determined X-ray crystal structures of MnmM complexed with anticodon stem loop of tRNA^Gln^. The structures provide the molecular basis underlying the importance of U33-nm^5^s^2^U34-U35 as the key determinant for the specificity of MnmM.

## Graphical Abstract

**Graphical Abstract:**
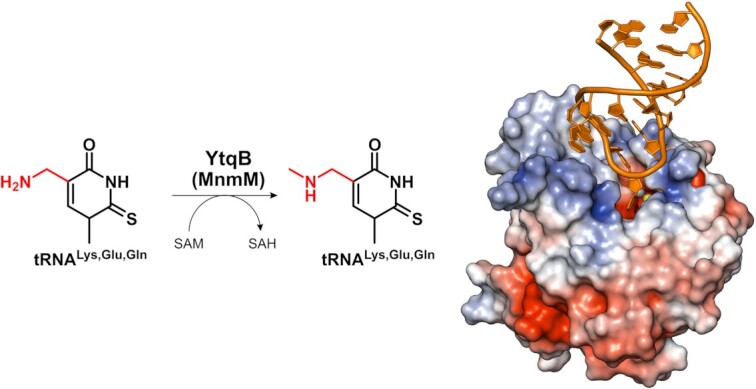
A novel methyltransferase MnmM, formerly known as YtqB, has been identified to be involved in the biosynthesis of mnm5s2U in tRNAs of Gram-positive bacteria and plants.

## INTRODUCTION

Ribonucleotides in nature undergo various post-transcriptional modifications. Thus far, >150 unique kinds of modifications have been identified, most of which are found in transfer RNA (tRNA) (1–3). Posttranscriptional modifications of tRNA are important for efficient and accurate aminoacylation by tRNA synthetases (4,5) and translation on the ribosome (4,6). The biological significance of certain tRNA modifications has been demonstrated in the regulation of cellular growth, metabolic control under stress conditions, and molecular signaling (7–9). In particular, the 5′-nucleotide of the anticodon, also known as wobble nucleotide (position 34 of tRNA), is most frequently targeted for modification; e.g. nearly 50% of *Escherichia coli* tRNA contain a modified nucleotide at the wobble position (10–13). The base-pairing interactions between the wobble nucleotide and the 3′-nucleotide of the codon are relatively more flexible than those in Watson–Crick pairs, which may result in the expansion or restriction of decoding specificity depending on the modification (14,15).

For example, hypermodified uridine 5-methylaminomethyl-2-thiouridine (mnm^5^s^2^U) induces conformational changes in the anticodon stem loop (ASL) region resulting in the stabilization of the stacking of nearby bases, reduction of error rate and frameshifting, and enhancement of translational efficiency (6,16–19). In addition, it has been shown that mnm^5^U confers decoding preferences towards NNG rather than NNA codons (20,21). Removal of both s^2^- and xm^5^-modifications resulted in the translational frameshift or reduced translational efficiency and caused pleiotropic phenotypes like synthetic lethality in growth, high sensitivity to pH, and translational defects in bacteria and eukaryotes (22,23).

**Figure 1.**
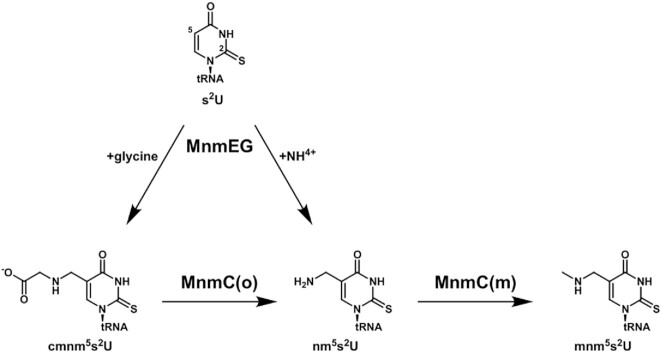
Biosynthetic pathway of mnm^5^s^2^U modification in *E*.*coli*. The s^2^U is modified to cmnm^5^s^2^U or nm^5^s^2^U by MnmEG complex with glycine or ammonium as a substrate, respectively. Then, MnmC(o) domain of bifunctional enzyme MnmC removes carboxymethyl group of cmnm^5^s^2^U to form nm^5^s^2^U. Finally, MnmC(m) domain methylates nm^5^s^2^U using SAM as methyl donor to generate mnm^5^s^2^U.

The biosynthetic pathway of mnm^5^(s^2^)U in *E. coli* has been thoroughly investigated (24); the methylaminomethyl group is added to the C5 atom of wobble uridine of Lys-, Glu-, Gln-, Gly-, and Arg-specific tRNA in sequential processes involving MnmE-MnmG complex and bifunctional enzyme MnmC (Figure 1). First, the MnmE–MnmG complex introduces the aminomethyl (nm-) or carboxymethylaminomethyl (cmnm-) group on the C5 of wobble uridine using ammonium or glycine as a substrate, respectively (25–27). The two-domain enzyme MnmC then catalyzes the last two steps of the pathway. A flavin adenine dinucleotide (FAD)-dependent oxidoreductase domain, MnmC(o), converts cmnm^5^(s^2^)U into nm^5^(s^2^)U. Subsequently, an *S*-adenosyl-L-methionine (SAM)-dependent methyltransferase domain, MnmC(m), methylates nm^5^(s^2^)U to form mnm^5^(s^2^)U (28–30). Independent of the C5 modifications, the C2 atom of the wobble uridine is subject to thiolation (s^2^U) or rarely to selenation (se^2^U) by MnmA or SelU, respectively (31–33).

Contrary to universally conserved *mnmE*-*mnmG* genes in bacteria (6), the *mnmC* gene has been found in proteobacteria and some species of spirochaetes only (28,34,35), but not in Gram-positive bacteria and plants. Interestingly, the presence of mnm-modified uridines has been reported in tRNAs of certain Gram-positive bacteria and plants (36–41). Modern liquid chromatography–mass spectrometry (LC–MS)-based analyses of cellular tRNAs have confirmed that the modifications are distributed in Gram-positive bacteria like *Bacillus subtilis* (42,43) and *Staphylococcus aureus* (44) and plant chloroplasts such as *Arabidopsis thaliana* (45) and *Oryza sativa* (46). These studies suggest that an MnmC-like activity must be present in those organisms. Moreover, the *in vitro* activity of mnm^5^s^2^U formation was demonstrated using *B. subtilis* lysates (34). The precise identity of the MnmC-like enzyme(s), however, has been elusive to date.

Here, we report that *ytqB* (renamed as *mnmM*) encodes the MnmC-like methyltransferase, which was previously annotated as a putative rRNA methylase. The formation of mnm^5^s^2^U from nm^5^s^2^U was verified both *in vivo* and *in vitro* by MnmM from *B*. *subtilis*. Additionally, our biochemical assays demonstrated that the orthologs from *S*. *aureus*, *A*. *thaliana* and *O*. *sativa* were capable of producing mnm^5^s^2^U. Furthermore, a total of five crystal structures of MnmM were determined, two of which are complexed with anticodon stem–loop (ASL) of tRNA. These collective data strongly support that the MnmM is responsible for methylating nm^5^(s^2^)U to produce mnm^5^(s^2^)U in Gram-positives and plants and provide the molecular basis for the structure-function relationship of the enzyme in the wobble uridine modification.

## MATERIALS AND METHODS

### Biological resources

*E. coli* and *B. subtilis* strains were purchased from Keio collection (NBRP, Japan) and Bacillus Genetic Stock Center (BGSC, USA), respectively. *E. coli* WT, *mnmC* and *mnmG* knockout strains are BW25113 (EcWT), JW5380-KC (Ec*ΔmnmC*) and JW3719-KC (Ec*ΔmnmG*), respectively; *B. subtilis* WT, *ytqB* and *mnmG* knockout strains are 1A1 (BsWT), BKK30490 (Bs*ΔytqB*) and BKK41010 (Bs*ΔmnmG*), respectively.

### Cloning and protein expression

*ytqB* gene from *B. subtilis* was amplified by polymerase chain reaction (PCR) from the genomic DNA of *B. subtilis* strain 168. The gene fused with a C-terminal His-tag was cloned using pLATE31 vector (Cat# K1261, Thermo Scientific) following the method provided by the manufacturer. *ytqB* genes from *Staphylococcus aureus, Arabidopsis thaliana*, and *Oryza sativa* were codon-optimized and synthesized by IDT (USA). For the plant orthologs, truncated constructs excluding the chloroplast signal peptide were used; 53–274 residues for *at*YtqB (*at*MnmM) and 48–260 residues for *os*YtqB (*os*MnmM) (47). The specific information of the proteins and primers used in this study is summarized in Supplementary Table S1-S2, respectively. Sequences of cloned genes were confirmed by Macrogen, Korea. For the heterologous expression of the proteins, the plasmids were used to transform *E. coli* BL21(DE3). The cells were incubated in an LB medium supplemented with 100 μg/ml ampicillin at 37°C while shaking at 160 rpm until OD_600_ reaches 0.4–0.8. Protein expression was induced by the addition of isopropyl β-d-1-thiogalactopyranoside (IPTG) to a final 0.1 mM concentration. Then, the flasks were moved to 20°C and incubated overnight while shaking at 160 rpm. The cells were pelleted by centrifugation at 14 372 rcf and 4°C for 10 min. The pellets were washed with buffer A (30 mM Tris–HCl pH 7.5, 150 mM NaCl, and 10% v/v glycerol) and re-pelleted by centrifugation at 3214 rcf and 4°C for 30 min. The washed pellets were stored at −86°C until protein purification. Cell pellets were thawed on ice and resuspended in 35 ml of buffer B (50 mM Tris–HCl pH 7.5, 500 mM NaCl, 10% v/v glycerol, and 50 mM imidazole) supplemented with 1 mg/ml lysozyme and 2 mM 1,4-dithiothreitol (DTT). The resuspended cells were incubated on a 3D rocker at 20°C for 20 min. The cells were lysed by sonication with 5 s pulse and 25 s rest for 10 cycles at 50% amplitude. The cell lysates were cleared by centrifugation at 21 672 rcf and 4°C for 30 min. The supernatants were filtered through a 0.2 μm pore size syringe filter and applied onto HisTrap HP 5 ml column (Cytiva, USA) pre-chilled with buffer B. His-tagged protein was eluted by applying imidazole gradient to 500 mM concentration. The eluted fractions were further purified by size exclusion chromatography (SEC) column (HiLoad 16/600 Superdex 75 pg, Cytiva) pre-equilibrated with buffer A supplemented with 5 mM 2-Mercaptoethanol (βME). The eluted fractions were collected, flash-frozen in liquid nitrogen (LN2), and stored at −86°C. Typical yields for pure *bs*YtqB (*bs*MnmM) or *sa*YtqB (*sa*MnmM) were 125 mg or 100 mg per liter of the medium, respectively. For *at*YtqB (*at*MnmM) and *os*YtqB (*os*MnmM), nearly 10 mg of pure proteins were obtained per one liter of culture.

### Bacterial bulk tRNA extraction

Methods for bulk extraction of tRNA from bacteria cells were modified from Kohrer *et al.* (48). Briefly, a single colony of an *E. coli* strain was inoculated in 500 ml of Luria-Bertani (LB) media and incubated overnight at 37°C while shaking at 160 rpm. Cell pellets were collected by centrifugation at 14 372 rcf and 4°C for 10 min and gently resuspended in 15 ml of buffer C (0.3 M sodium acetate pH 4.5 and 10 mM ethylenediaminetetraacetic acid (EDTA)). To permeate tRNA from the cell, 16.7 ml of acid-saturated phenol was added and gently rocked for 30 min at 4°C. The water layer was separated from the phenol layer by centrifugation at 3214 rcf and 4°C for 30 min and transferred to a new 50 ml conical tube. To precipitate tRNA, 2.5 volume of ethanol was added and overnight incubated at −20°C. tRNA pellets were collected by centrifugation at 3214 rcf and 4°C for 30 min. To wash out residual phenol content, the pellets were thoroughly washed with 70% ethanol. Residual ethanol was removed by drying the pellets in a 60°C oven for 10 min. The extracted bulk tRNA was resuspended in RNA-free water, flash-frozen in LN2, and stored at −86°C until used. tRNA extraction from *Bacillus subtilis* strains was performed as that of the methods for *E. coli* except for the addition of the lysozyme digestion step after the resuspension of cell pellet in buffer C; i.e. the resuspended cells were mixed with 1 mg/ml of lysozyme and incubated for 30 min at 37°C while shaking at 160 rpm.

### Gene complementation assay

For the complementation assay in *E. coli*, the *ytqB* gene from *B. subtilis* was cloned into the pQE30 vector using Ligation Independent Cloning (LIC) cloning. For the assay in *B. subtilis*, the genes were cloned into pHT01 (Mobitec) via *in vivo* cloning (49). Briefly, PCR amplified gene fragments were mixed with a linearized vector treated with BamHI and XmaI. The mixtures were directly used for DH5α transformation. Sequences of the cloned genes were confirmed by Macrogen, Korea. For complementation of *ec*MnmC, *E. coli ΔmnmC* cells were transformed with pQE30-*ytqB* vector. Colonies grown in LB agar plate with 100 μg/ml ampicillin and 50 μg/ml kanamycin were selected for tRNA extraction. To complement *B. subtilis ytqB* activity, the pHT01-*ytqB* vector was used to transform *B. subtilis ΔytqB*. Chemically competent *E. coli* or *B. subtilis* strains were generated using methods described in Chan *et al.* (50) or Anton *et al.* (51), respectively. Colonies grown in LB agar plates with 5 μg/ml kanamycin and 5 μg/ml chloramphenicol were selected for tRNA extraction. The selected colonies were used for phenol extraction of tRNA as described above. Subsequently, extracted tRNA was digested into nucleosides, and analyzed by HPLC and LC–MS as described below.

### *In vitro* assay

For *bs*YtqB and *sa*YtqB assays, 40 μg of renatured bulk tRNA was mixed with purified 1 μM enzyme and 0.1 mM SAM in 28 μl reaction buffer (10 mM Tris–HCl pH 7.5, 50 mM NaCl, and 3.3% v/v glycerol). For *os*YtqB and *at*YtqB assays, identical condition except for 20 μM YtqB was used. The reaction was performed in a water bath equilibrated overnight at 37°C. Next, tRNA was digested into nucleosides and analyzed by LC–MS as described below. Time-dependent activities of *bs*YtqB mutants were measured similarly with minor modifications: enzyme concentration was reduced to 0.2 μM, pH of SAM and tRNA were adjusted using 1.5–6 mM NaOH prior to the reaction, and reactions were carried out for 0–10 min at 25°C.

### tRNA hydrolysis and HPLC analysis

tRNA was hydrolyzed into nucleosides based on the method described in Jora *et al.* (52) with minor modifications. 40 μg of bulk tRNA was denatured at 95°C for 5 min, then renatured at room temperature. The hydrolysis reaction was performed in a one-pot method with a 40 μl volume. The tRNA was mixed in buffer D (50 mM ammonium acetate pH 6.0, 5 mM ZnCl_2_, and 10 mM MgCl_2_) with 0.2 U of Nuclease P1 (Cat# N8630, Sigma-Aldrich), 0.2 U of Phosphodiesterase I (PDE I, Cat# P3243, Sigma-Aldrich), and 2 U of FastAP (Cat# EF0651, Thermo Scientific). The mixture was incubated overnight at 37°C and flash-frozen until analysis. Prior to HPLC analysis, the reactants were thawed on ice and centrifuged for 10 min at 17 000 rcf and 4°C. A 20 μl of the supernatants was subjected to a reversed-phased HPLC column (Develosil® RP-Aqueous C30, 140Å, 4.6 × 150 mm, 5 μm, Nomura Chemical) equipped with 1260 Infinity (Agilent). For the analysis of nucleosides derived from *B. subtilis*, a 10 mM ammonium acetate pH 4.5 in water/methanol 97:3 (v/v) or 2:98 (v/v) were used as solvent A or B, respectively. Gradient program was followed: initial column equilibration using 0% B for 30 min; ramping up to 100% B at 31 to 35 min; hold for 35.1 to 45 min at 0% B for re-equilibration. For the nucleosides from *E. coli*, a 10 mM ammonium acetate pH 5.0 in water/methanol 95:5 (v/v) was used as solvent A. Gradient program was also modified: initial column equilibration using 0% B for 20 min; ramping up to 16.1% B at 22.5 min and 100% at 24 to 28 min; hold for 28.1 to 37.5 min at 0% B for re-equilibration. The detector wavelength was set to 314 nm to maximize the signal-to-noise ratio of thiolated uridine. The HPLC results were plotted using OriginPro 2021 (OriginLab).

### LC–MS analysis of nucleosides

The samples were separated by the reversed-phase column (ZORBAX RR Eclipse Plus C18, 95Å, 4.6 × 100 mm, 3.5 μm, Part# 959963–902, Agilent) equipped with UltiMate 3000 (Thermo Scientific). The column temperature was set to 20°C. The flow rate was set to 0.4 ml/min. An aliquot of 15 μl of the samples was injected. A 0.1% v/v formic acid in water or acetonitrile was used as solvent A or B, respectively. Gradient program was as following: ramping up 1–12% B over 17.5 min; 70% B at 20–25 min; hold for 25.1 to 55 min at 1% B for re-equilibration. Mass spectrometry was performed by LTQ Orbitrap XL (Thermo Scientific). HPLC elution was ionized by electrospray ionization (ESI). Ionized molecules were analyzed under positive mode. LC–MS parameters were as following: mass range, 100–600 *m*/*z*; activation type, higher energy CID (HCD); collision energy, 35 V; activation time, 30 ms; sheath/aux/sweep gas flow rate, 40/10/1 arbitrary unit; capillary temperature, 350°C; source/capillary/tube lens voltage, 3000/21/60 V. Raw format data file was extracted as mzML format and analyzed by MZmine2 (53).

### Crystallization and structural determination

All crystallization experiments were performed via sitting drop vapor diffusion methods at room temperature and the crystallization mixtures were equilibrated against 60 μl mother liquor except for *bs*YtqB-SAM-ASL, as described below in more detail. For *S*-adenosyl-l-homocysteine (SAH)-bound *bs*YtqB crystals, we initially attempted to co-crystallize with SAM and full-length *bs*Gln-tRNA transcribed *in vitro*. However, electron densities corresponding to tRNA were absent in the initial map, thus it was not included in further model building processes. SAH was modeled in place of SAM, because of the lack of electron densities near the *S*-methyl group, which was most likely caused by hydrolysis over an extended period of crystallization trial (∼5 months) before X-ray diffraction experiment. The *bs*YtqB protein solution (10 mg/ml) was mixed with tRNA and ligands to final concentrations of 0.14 mM *bs*YtqB, 0.21 mM *bs*Gln-tRNA, 1 mM SAM, and 5 mM MgCl_2_ in buffer A. An aliquot of 0.8 μl of the mixture was mixed with an equal volume of crystallization mother liquor. Crystals were formed in mother liquor composed of 0.1 M HEPES pH 7.5 and 25% w/v polyethylene glycol (PEG) 3350. The crystals were cryo-protected by mother liquor supplemented with 20% w/v glycerol prior to flash-freezing in liquid nitrogen. For the crystallization of *bs*YtqB-SAM-ASL, *bs*Gln-tRNA ASL (17 nt, ACGGACUUUGACUCCGU) was purchased from IDT. The protein solution was mixed with *bs*Gln-tRNA ASL and ligands to final concentrations of 0.14 mM *bs*YtqB, 0.28 mM *bs*Gln-tRNA-ASL, 1 mM SAM, and 1 mM MgCl_2_ in buffer A. The crystals were obtained by hanging drop vapor diffusion method at 20°C, and 1.5 μl mixture was added to the equal volume of reservoir mother liquor containing 0.2 M ammonium acetate, 0.1 M sodium citrate tribasic dihydrate pH 5.6, 30% w/v PEG 4,000, and 3% w/v trimethylamine N-oxide dihydrate. The crystals were cryo-protected by mother liquor supplemented with 20% w/v glycerol before flash-freezing in liquid nitrogen.

Rhomboid-shaped apo *sa*YtqB crystals were formed by mixing 0.8 μl of 11.2 mg/ml (0.51 mM) protein solution with an equal volume of mother liquor (0.2 M sodium acetate trihydrate, 0.1 M Tris–HCl pH 8.5, and 30% w/v PEG 4,000). The crystals were cryo-protected by mother liquor supplemented with 30% w/v PEG 4000 before flash-freezing in liquid nitrogen. Rod-shaped SAM-bound *sa*YtqB crystals were formed by mixing 0.8 μl of a cocktail solution containing 7.50 mg/ml (0.34 mM) protein, 1.39 mM SAM, and 5 mM MgCl, with an equal volume of mother liquor (0.2 M lithium sulfate monohydrate, 0.1 M Bis–Tris pH 6.5, and 25% w/v PEG 3350). The crystals were flash-frozen in liquid nitrogen without cryo-protectant. Since the ASL sequences of *bs*Gln-tRNA are identical to those of *sa*Gln-tRNA, we used the *bs*Gln-tRNA ASL for the co-crystallization with *sa*YtqB. Rhomboidal ASL/SAM-bound *sa*YtqB crystals were formed by mixing 0.8 μl of crystallization cocktail (5.09 mg/ml (0.23 mM) protein, 2.49 mg/ml (0.46 mM) *bs*Gln-tRNA ASL, 1 mM SAM, and 5 mM MgCl_2_) with an equal volume of mother liquor containing 0.2 M magnesium acetate tetrahydrate, 0.1 M sodium cacodylate trihydrate pH 6.5, and 20% w/v PEG 8,000. The crystals were flash-frozen in liquid nitrogen without cryo-protectant.

All X-ray diffraction data were collected under cryogenic conditions using the Eiger X 9 M detector at Pohang Accelerator Laboratory (PAL; South Korea) beamline 5C (Supplementary Table S3). The data were indexed, integrated, and scaled by XDS (54) or autoPROC (55). An anisotropic resolution cut-off was applied by STARANISO (56). In the case of the *bs*YtqB-SAM-ASL structure, isotropic cut-off was applied by Aimless (57). To solve the phase problem, molecular replacement was performed using the SAM-bound *bs*YtqB structure (PDB: 4POO) as a search model using MOLREP (58). Initial model building was enabled by ModelCraft (59) and PDB_REDO server (60). The final model was polished by iterative cycles of real space model building using Coot (61) and refinement by REFMAC5 (62) and Phenix.refine (63). The structures were visualized by PyMOL (64).

## RESULTS

### Identification of the mnmC-like methyltransferase gene via comparative genomics

**Figure 2.**
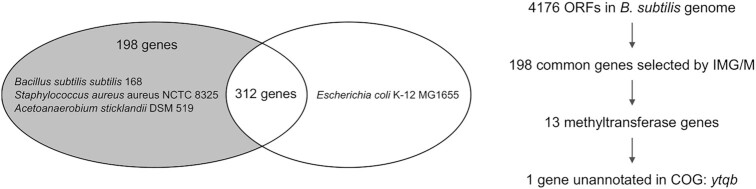
Comparative genomics searching for MnmC-like enzyme(s). (Left) A Venn diagram representing the distribution of 198 genes commonly present in *Bacillus subtilis* subtilis 168, *Staphylococcus aureus* NCTC 8325, and *Acetoanaerobium sticklandii* DSM 519, but absent in *Escherichia coli* K-12 MG1655. (Right) A scheme describing the process for identifying the candidate gene(s) responsible for 5-mnm modification in this study. ORF, open reading frame; IMG/M, Integrated Microbial Genomes & Microbiomes system; COG, Clusters of Orthologous Genes.

To search for candidate genes expressing MnmC-like enzymes, we applied comparative genomics (65), which was used for the identification of various novel tRNA-modifying enzymes including epoxyqueuosine reductase QueH, tRNA U34 hydroxylase TrhO, and tRNA 3-amino-3-carboxypropyltransferase TapT (66–68). Based on the fact that the presence of mnm^5^-modifications has been confirmed in three Gram-positive bacteria, *B*. *subtilis* (42,43), *S*. *aureus* (44) and *Acetoanaerobium sticklandii* (41), first we sought to identify the gene(s) conserved in common among those species but absent in *E. coli* using Phylogenetic Profiler tool in IMG/M database (69) (Figure 2). We used ‘Single genes’ tool under ‘Find Genes’> ‘Phylogenetic Profiler’ tab and selected *Bacillus subtilis subtilis 168* in the ‘Step 1. Find Genes In’ menu. *Staphylococcus aureus aureus NCTC 8325* and *Acetoanaerobium sticklandii DSM 519* were selected for ‘Step 2a. With Homologs In’ and *E. coli K-12 MG1655* for ‘Step 2b. Without Homologs In’. The default values for similarity cutoffs were used including the *E*-value (10^−5^) and maximum percent identity (30%). As a result, a total of 198 genes were obtained in these criteria, which are summarized in Supplementary Table S4. Initially, we expected to find a gene that encodes a single polypeptide, in which oxidoreductase and methyltransferase domains are fused together analogous to *E. coli* MnmC, or an operon that expresses the two enzymes. However, we were unable to find the genes satisfying the aforementioned condition. Based on Clusters of Orthologous Genes (COG) database (70), 13 out of those 198 genes were classified as methyltransferase and none was annotated as FAD-dependent oxidoreductase. Upon further analysis through COG database, three putative methyltransferases initially identified from the IMG/M database, YabB, YabC and YpsC, were in fact tRNA1(Val) (adenine(37)-N6)-methyltransferase, 16S rRNA 2′-*O*-ribose C1402 methyltransferase, and 23S rRNA m2G2445 methyltransferase, respectively. Of interest, no specific function was assigned to one methyltransferase gene, *ytqB*, the product of which was labeled as a putative rRNA methylase in the UniProt database (71). Although the structure of this protein was reported previously (72), the exact cellular function has not been characterized to date. Thus, we hypothesized that *ytqB* might encode an MnmC-like methyltransferase.

### Gene complementation assay of *B*.*subtilis* ytqB

**Figure 3.**
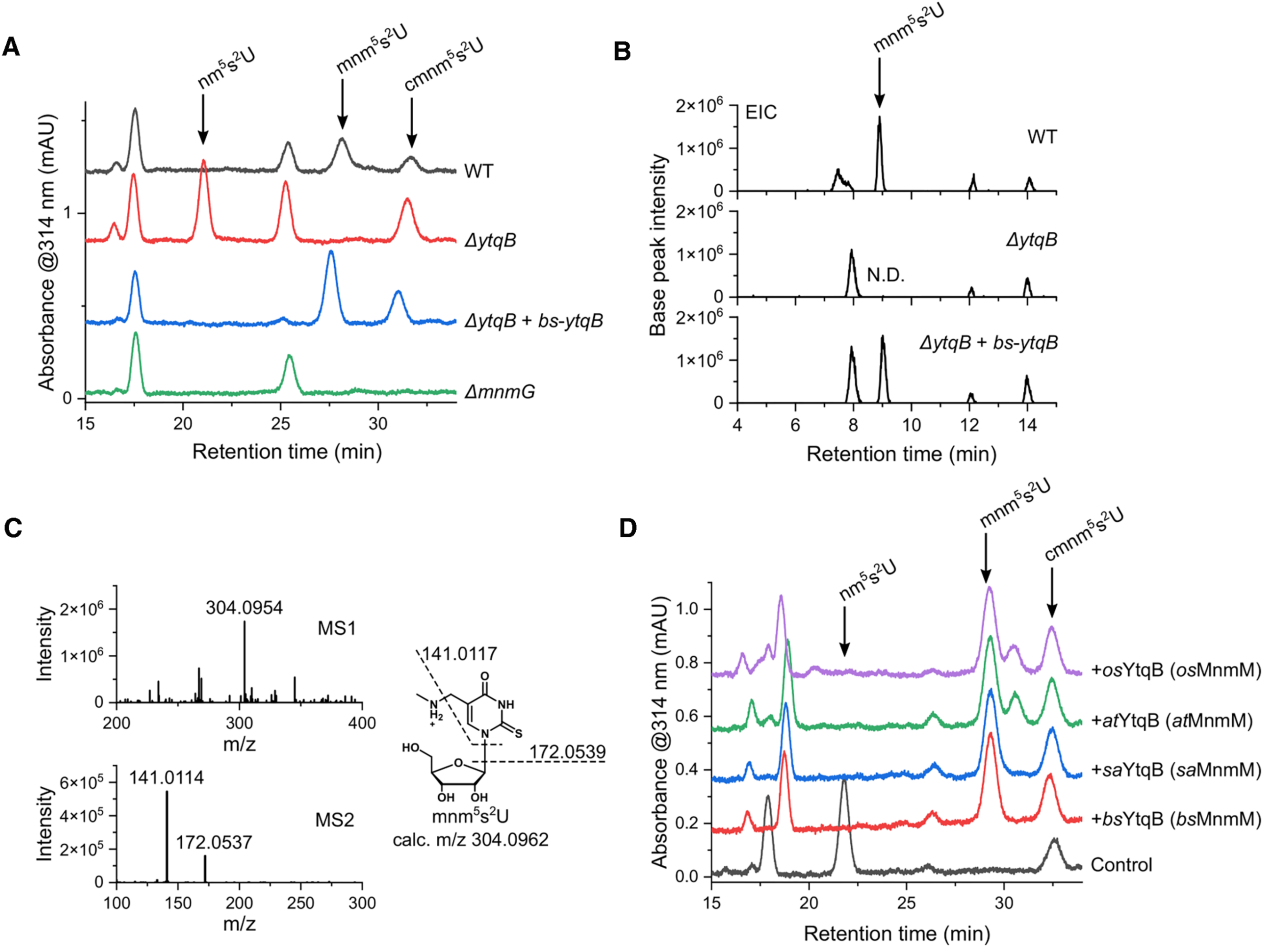
Gene complementation and *in vitro* assays of *B. subtilis* YtqB and its orthologs. (**A**) HPLC analyses of gene complementation experiments showing the xm^5^s^2^U contents of bulk tRNA extracted from *B*. *subtilis* wild-type, *ΔytqB*, *ΔytqB* transformed with a plasmid pHT-*bs*-*ytqB*, and *ΔmnmG* strains. (**B**) Extracted ion chromatograph (EIC) corresponding to an *m*/*z* value of mnm^5^s^2^U (*m*/*z* 304.09617 ± 0.01) from *B*. *subtilis* wild-type, *ΔytqB*, and *ΔytqB* transformed with a plasmid pHT-*bs*-*ytqB* strains. N.D., not detected. (**C**) Representative MS1 and MS2 spectra of mnm^5^s^2^U and fragmentation patterns of mnm^5^s^2^U. (**D**) HPLC analyses of *in vitro* methylation assay of heterologously expressed YtqBs of various species with nm^5^s^2^U-containing bulk tRNA extracted from *B. subtilis ΔytqB* strain. Reaction samples without enzyme was used as a negative control (control). bs, *B. subtilis*; sa, *S. aureus*; at, *A. thaliana*; os, *O. sativa*.

To test whether YtqB acts as an MnmC-like methyltransferase in *B. subtilis*, gene complementation assays were performed using a *B*. *subtilis* variant lacking *ytqB*. The composition of bulk tRNAs from *B. subtilis* cells was analyzed via high-performance liquid chromatography (HPLC) (Figure 3A). Of interest, mnm^5^s^2^U was not detectable in the tRNA sample from *ytqB*-deficient cells, whereas the enrichment of nm^5^s^2^U was observed instead, which was absent in the tRNA sample derived from the wild-type *B. subtilis*. When the mutant strain was transformed with a plasmid carrying the *ytqB* gene, the formation of the mnm^5^s^2^U was restored while nm^5^s^2^U became essentially undetectable, similar to the wild-type. For comparison, the bulk tRNA sample prepared from *ΔmnmG B. subtilis* was analyzed and neither nm^5^s^2^U nor mnm^5^s^2^U was detectable in our assay condition. We confirmed the results from the HPLC-based analysis with LC–MS, where the mass-over-charge ratio (*m/z*) corresponding to that of mnm^5^s^2^U was clearly detected in the samples of wild-type and *ytqB*-deficient cells complemented with the expression plasmid, but not in the sample of *ytqB*-deficient cells (Figure 3B). Additionally, the MS/MS spectrum was consistent with the fragmentation of mnm^5^s^2^U as shown in Figure 3C, validating our peak assignment. As Moukadiri *et al.* demonstrated that the cellular extracts of *B. subtilis* restored the loss of mnm^5^s^2^U in tRNA derived from *mnmC*-deficient *E. coli* (34), we tested whether *B. subtilis ytqB* could substitute *mnmC* in the synthesis of mnm^5^s^2^U in *E*. *coli* cells (Supplementary Figure S1A). Similar to the results of the complementation experiment with *ytqB*-deficient *B. subtilis*, the formation of mnm^5^s^2^U was rescued when the mutant *E. coli* cells were transformed with a plasmid harboring the *B*. *subtilis ytqB* gene.

### *In vitro* methyl transfer reaction of recombinantly expressed *B*.*subtilis* YtqB

Next, we examined the *in vitro* activity of *B. subtilis* YtqB in the conversion of nm^5^s^2^U to mnm^5^s^2^U. Hypomodified bulk tRNAs containing nm^5^s^2^U were isolated from *ytqB*-deficient *B*. *subtilis* or *mnmC*-deficient *E. coli* strain and used as a substrate for enzymatic reactions. Recombinant *B. subtilis* YtqB (*bs*YtqB, UniProt ID: O34614) was heterologously overexpressed and purified from *E. coli*. The isolated bulk tRNAs were mixed with the methyl donor SAM and the purified recombinant *bs*YtqB and analyzed via HPLC (Figure 3D and Supplementary Figure S1B). The enzyme was able to form mnm^5^s^2^U when tRNA from either *ΔytqB B. subtilis* or *ΔmnmC E*. *coli* was used, in line with the results of gene complementation assays. Therefore, our *in vitro* assay results indicate that YtqB is necessary and sufficient for mnm^5^s^2^U synthesis at the wobble position of the cognate tRNAs. Combined with the genetic and *in vivo* data, these results strongly support that YtqB is the **Mnm**C-like **m**ethyltransferase and we propose to rename it to MnmM.

### *In vitro* activities of MnmM orthologs

Based on the previous reports that 5-mnm-modified uridines were also found in tRNA from the Gram-positive bacterium *S. aureus* and plants *A. thaliana* and *O. sativa* (44–46), we hypothesized that an enzyme homologous to *bs*MnmM might be present in those organisms. The MnmM ortholog in *S. aureus* (*sa*MnmM, UniProt ID: Q2FXG9) shares 52% of amino acid sequence identity with *bs*MnmM, whereas two orthologs from *A. thaliana* (*at*MnmM, UniProt ID: Q8GUP2) and *O. sativa* (*os*MnmM, UniProt ID: Q0DGU2) were 36% and 38% identical to *bs*MnmM, respectively (Supplementary Figure S2). To examine the nm^5^s^2^U methylation activity of these proteins, the purified recombinant enzymes were used for *in vitro* assays. The assay results confirmed that heterologously expressed *sa*MnmM, *at*MnmM, and *os*MnmM were capable of methylating the hypomodified tRNA substrates derived from *ΔytqB B. subtilis* to yield mnm^5^s^2^U (Figure 3D).

### X-ray crystal structures of MnmM

**Figure 4.**
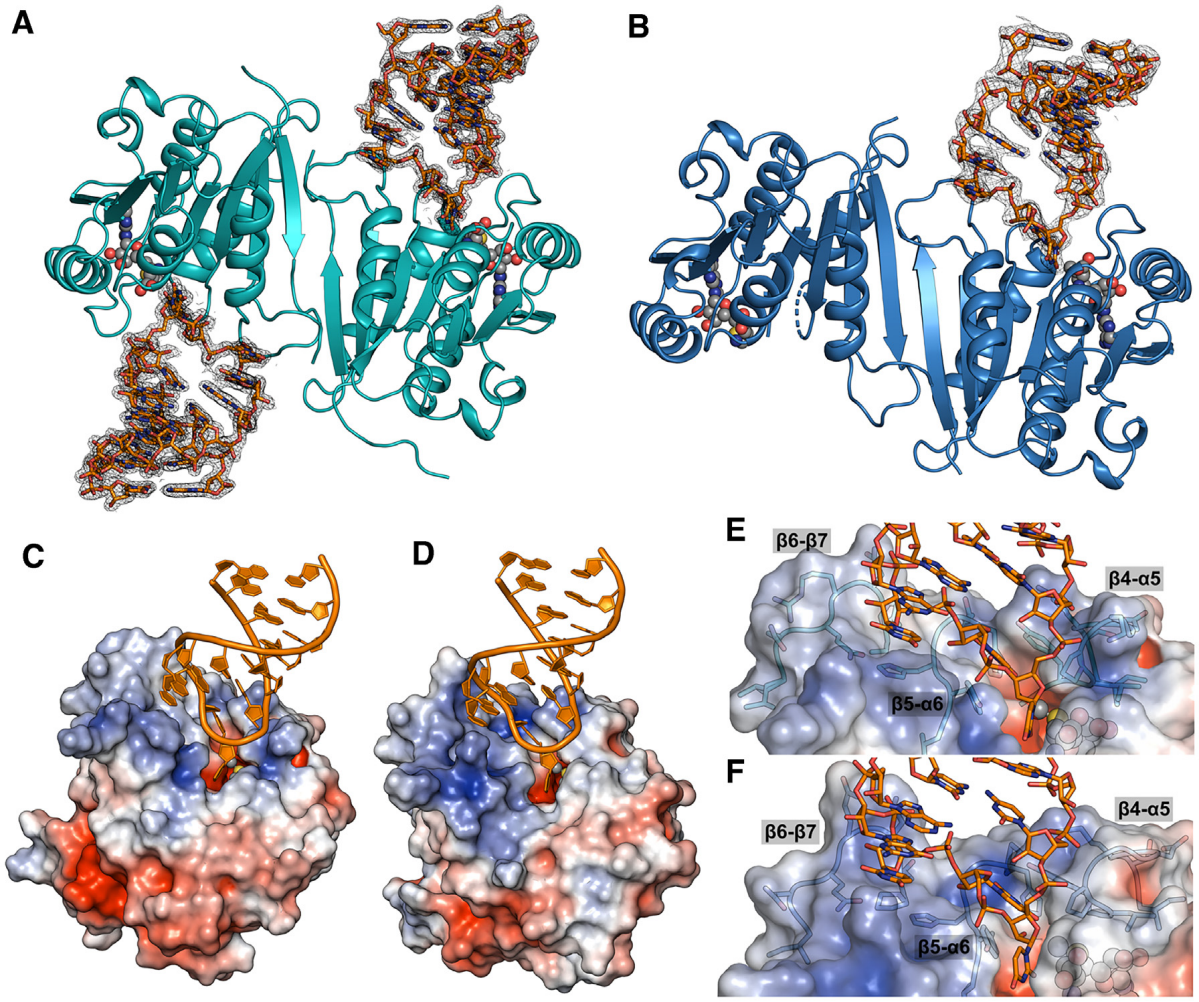
Overall view of tRNA-MnmM interactions. The structure of MnmM-SAM-ASL complex. MnmM dimer is shown in cartoons, ASL in sticks, and SAM in balls. Fourier difference maps (2*Fo* – *Fc*) contoured at 1.0-σ show electron densities around ASL bound to (**A**) *sa*MnmM (cyan) and (**B)***bs*MnmM (navy). Electrostatic potential (blue is positive, red is negative, and white is neutral) is mapped on the surfaces of *sa*MnmM-SAM-ASL (**C**, **E**) and *bs*MnmM-SAM-ASL (**D**, **F**).

Although apo and SAM-bound structures of the *bs*MnmM are available (72), how this enzyme interacts with the substrate tRNA is completely unknown. To interrogate detailed molecular interactions between MnmM and tRNA, we attempted to co-crystallize MnmM orthologs in the presence of a cofactor and an unmodified 17-mer ASL of Gln-specific tRNA for X-ray crystallographic studies. After optimization of initial growth conditions, several diffraction-quality crystals were obtained in various forms with *sa*MnmM and *bs*MnmM samples. A total of five structures were solved to a resolution ranging from 1.17 Å to 2.90 Å (Supplementary Table S3); *i.e*. apo *sa*MnmM (*sa*MnmM-apo, PDB: 8H1A), SAM-bound *sa*MnmM (*sa*MnmM-SAM, PDB: 8H27), ASL- and SAM-bound *sa*MnmM (*sa*MnmM-SAM-ASL, PDB: 8H1B), SAH-bound *bs*MnmM (*bs*MnmM-SAH, PDB: 8H0T), and ASL- and SAM-bound *bs*MnmM (*bs*MnmM-SAM-ASL, PDB: 8H0S). The overall conformations of all five structures are highly similar to one another with root-mean-square deviation (RMSD) of 0.35–1.30 Å, in which both *sa*MnmM and *bs*MnmM exhibit an analogous dimeric conformations. (Figure 4 and Supplementary Figure S3). The oligomeric state of *sa*MnmM in solution was examined by size exclusion chromatography and the formation of the dimer was confirmed as the *B. subtilis* ortholog (72). The binding of SAM or SAH does not significantly alter the global conformation of MnmM protein in general (RMSD values, 0.35–0.67 Å), but sometimes induces the organization of the β4-α5 and β5-α6 loops, which are typically disordered when ASL is not bound (Supplementary Table S5).

### Recognition of anticodon stem-loop at the tRNA binding surface of MnmM

**Figure 5.**
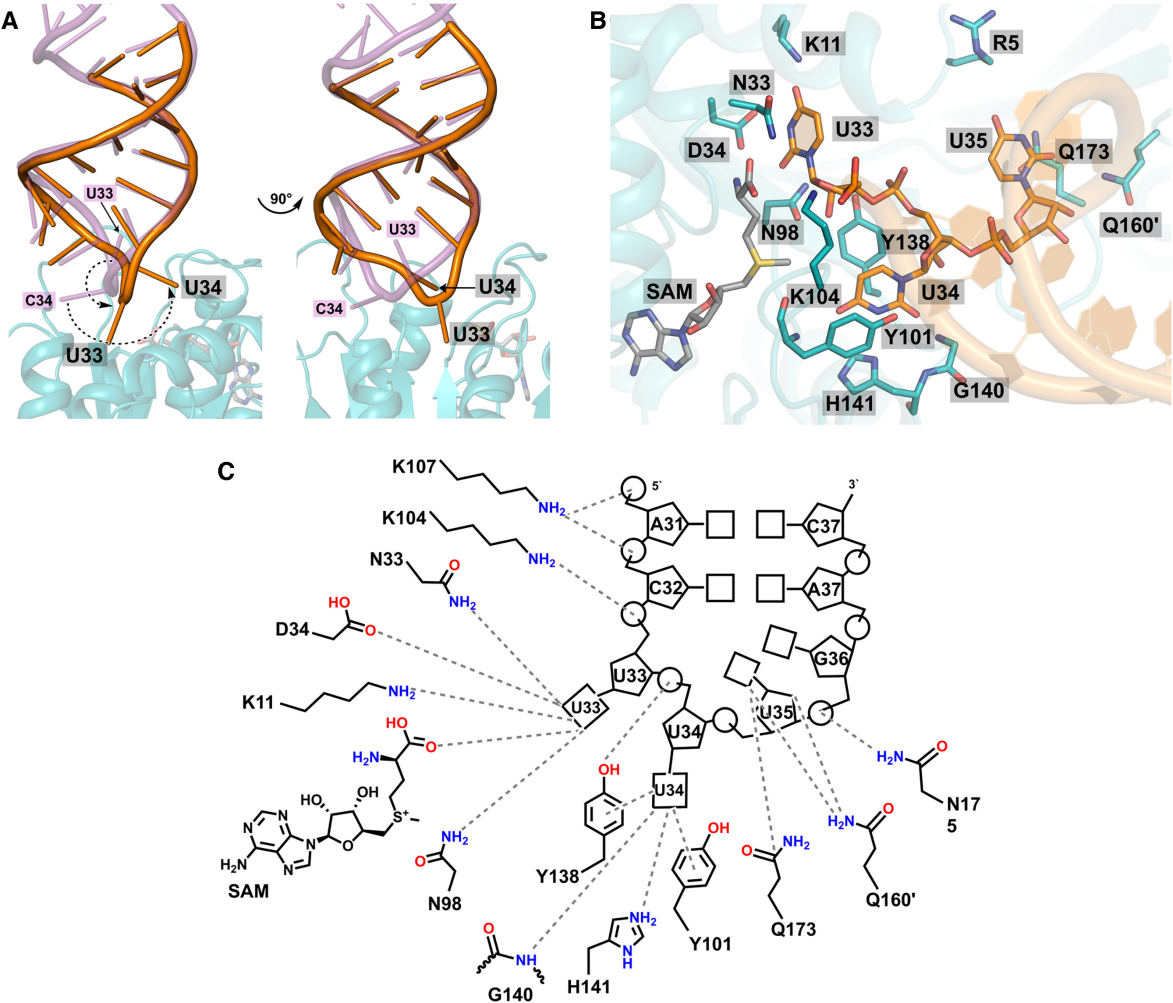
Base flipping of U33 and U34 in anticodon loop upon binding to *sa*MnmM. (**A**) Active site of *sa*MnmM-SAM-ASL highlighting the interactions with U33, U34, and U35 of bound ASL (carbon atoms in orange) and SAM (carbon atoms in grey), compared with a ‘canonical’ tRNA (4V7M, purple). Bases of both U33 and U34 are flipped-out in the ASL-*sa*MnmM complex structure. (**B**) Close-up of the active site showing the molecular interactions among the anticodon loop of tRNA, *sa*MnmM and SAM. (**C**) Schematic diagram of interactions between the ASL with amino acid residues of *sa*MnmM. Oxygen is shown in red, nitrogen in blue, and sulfur in yellow.

Structures of ASL-bound complex display a different stoichiometry of two macromolecules in the complexes; *i.e*. one tRNA molecule is bound to the dimer of *bs*MnmM, whereas two are complexed with that of *sa*MnmM (Figure 4A, B**)**. Otherwise, highly similar intermolecular interactions between MnmM and tRNA ASL are observed in both structures. Major interactions with tRNA occur on the positively charged cleft of the enzyme formed by β4–α5, β5–α6, and β6–β7 loops (Figure 4C–F). A survey of each protomer in the structures of MnmM reveals that β4–α5 and β5–α6 loops tend to be disordered without ASL, whereas the β6–β7 loop is fully ordered in all structures regardless of ligand binding (Supplementary Figure S4 and Supplementary Table S5). In both *sa*MnmM-SAM-ASL and *bs*MnmM-SAM-ASL structures, the enzyme contacts tRNA mainly in the single-stranded loop region spanning A31 through G36 (Figures 5 and Supplementary Figure S5). The anticodon loop of the MnmM-bound tRNA undergoes a substantial conformational change from the canonical form, in which nucleobases are stacked on one another as usually observed in the structures of free- or ribosome-bound tRNA (73). Specifically, the backbone conformation of the anticodon loop around U33–U34–U35 becomes more blatant compared to that of unbound tRNA, where the bases of U33 and U34 are flipped out towards the active site, facilitating the access of the target nucleobase to the enzyme (Figures 5A, B and Supplementary Figure S5A, B).

The uracil base of U33, a universally conserved nucleotide among all tRNA, forms an extensive hydrogen bonding network with the side chain of K11 (K11), D34 (D34), and N98 (N101) in the structures of *sa*MnmM-SAM-ASL and *bs*MnmM-SAM-ASL (residues in parentheses) (Figure 5C and Supplementary Figure S5C). The 2′-hydroxyl of U33 is contacted with N98 (N101) and the phosphate interacts with K104 in *sa*MnmM-SAM-ASL, which is absent in *bs*MnmM-SAM-ASL structure. Notably, U34 is recognized via π–π stacking, where the uracil ring is sandwiched between the side chains of Y101 (Y104) and Y138 (Y141). Moreover, the wobble base forms multiple hydrogen bonds with the protein; the N^3^ of U34 base forms a hydrogen bond with the side chain of H141 (H144), and additional ones are provided from the main chain amino group of G140 (G143) and Y101 (Y104) to the O^2^ and O^4^, respectively. The backbone phosphate of U34 participates in polar interactions with the side chain of Y138 (Y141). Together with U33 and U34, U35 is one of three residues in ASL involved in polar interactions with MnmM via its nucleobase. Unlike the other two, however, U35 maintains the stacked conformation conferring limited access to the protein, where the major polar interaction involving U35 base appears to be the amine-π stacking with the side chain of Q173 (Q176). Interestingly, Q160 (Q163) from the other protomer within the MnmM dimer contribute to the formation of hydrogen bonds with the O4′-oxygen of U35 and O2′-oxygen of ribose via their side chains. The U35 phosphate and G36 ribose interact with N175 in *sa*MnmM and Q177 and N178 in *bs*MnmM. Near the stem of tRNA, the side chain of K107 (K110) participates in the salt-bridge formation with the backbone phosphates of A31 and C32, and the main chain amine group of K104 (G107) provides an additional hydrogen bond to the phosphate of C32 (Supplementary Figure S6).

### Mutagenesis studies of key residues in tRNA binding and catalysis

**Figure 6.**
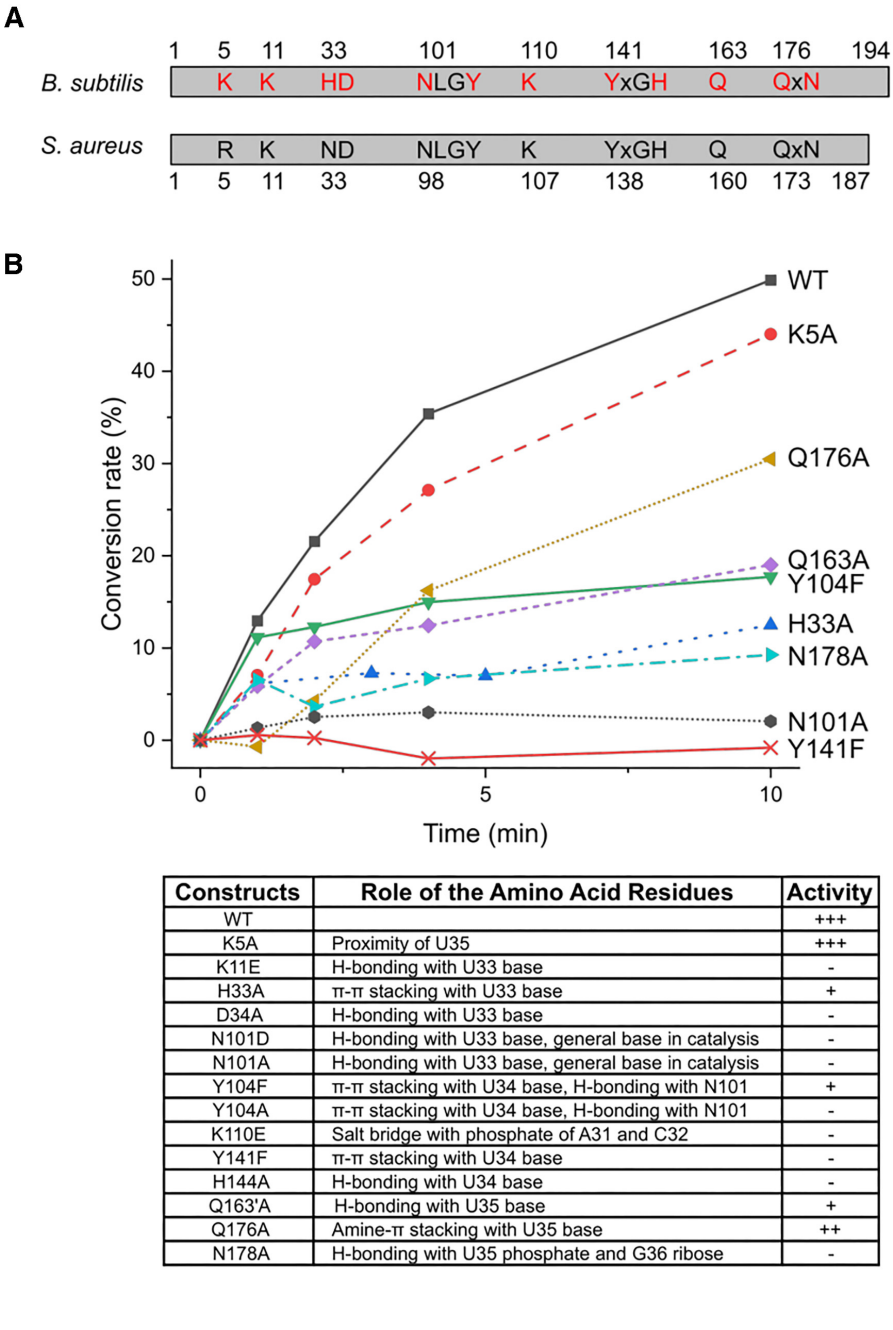
Mutagenesis assays of *bs*MnmM. (**A**) Conserved residues in *bs*MnmM and *sa*MnmM. The residues targeted for site-directed mutagenesis are colored in red. (**B)** The conversion rate between nm^5^s^2^U to mnm^5^s^2^U was plotted, using the wild-type and mutant MnmMs, nm^5^s^2^U-rich tRNA extracted from *B. subtilis ΔytqB*, and SAM. Activities of K11E, D34A, N101D, Y104A, K110E and H144A were very low as those of N101A and Y141F, and were excluded for clarity. Relative activities of all mutants are summarized in the bottom table, where +++ denotes the highest, ++ for modest, + for marginal and – for the lowest activity.

Next, site-directed mutagenesis was performed to examine the role of individual amino acid residues in tRNA modification, where target residues from *bs*MnmM were selected for mutation based on our structural information and conservation analysis (Supplementary Figures S2 and S7). Results from kinetic assays of *bs*MnmM mutants unveil the functional importance of the tRNA interacting residues identified in the structure (Figure 6). Particularly, when those amino acid residues interacting with the U33 and U34 were mutated, the enzymatic activities become nearly undetectable in our assay condition; e.g. K11E, D34A, N101D, N101A, Y104A, Y141F, and H144A. It is notable that when Y141, which sandwiches the base of U34 together with Y104 via π–π stacking interactions, was replaced with phenylalanine, the mutant protein was inactive, whereas Y104F retained approximately three-fold reduced activity compared to the wild-type enzyme. The activity was totally abolished when Y104 was changed to alanine, which is likely to have resulted from the loss of critical π–stacking interaction. The significance of this observation concerning the catalytic mechanism will be discussed in detail later. For amino acid residues interacting with U35, which include Q163, Q176 and N178, their alanine mutants displayed 20–60% activity compared to that of the wild-type. Therefore, the recognition of U35 by MnmM appears to be not as strict as that of U33 and U34. Alanine mutation of K5, which is positioned ∼3.8 Å away from the uracil base of U35, exhibited no effect on methylation activity. Meanwhile, K110E exhibited insignificant activity, indicating that the coulombic interactions with the phosphates of A31 and C32 are critical as well.

## DISCUSSION

### Discovery of novel enzymatic activities in mnm^5^-modification pathway

Although xm^5^s^2^U modifications and MnmEG pathway are present in most Gram-positive bacteria and plants, an MnmC ortholog is missing in those organisms. In this study, we discovered the MnmC-like methyltransferase MnmM by comparative genomics, verified the cellular and *in vitro* activities, and determined the tRNA-bound structures. However, the identity of MnmC(o)-like enzyme remains unknown in those organisms containing MnmM, which is currently under investigation in our laboratory. Phylogenetic distribution of *mnmM*, *mnmC(o)* and *mnmC(m)* illustrates that *mnmM* and *mnmC* are conserved in a mutually exclusive manner, where the former is mostly found in Firmicutes, Chlamydia, Mollicutes, and plants, and the latter in Proteobacteria, Bacteriodetes, Cyanobacteria, Spirochaetia, and Archaea (Supplementary Figure S8 and Supplementary Table S6). Notably, *mnmC(o)* is absent in Archaea, Bacteroides, and α-Proteobacteria. In most cases, bacteria containing *mnmC(m)* also include *mnmC(o)* in their genome (125 out of 213), yet the other species do not; e.g. α-Proteobacteria, Bacteroides, Cyanobacteria, and Archaea. These organisms with the orphan *mnmC(m)* gene are likely to encode an MnmC(o)-like enzyme and so are Firmicutes and plants that contain *mnmM*. Notably, the MnmC(o)-like activity was demonstrated in the cell lysates of *B. subtilis* using *E. coli* tRNA as a substrate *in vitro* (34). It will be interesting to identify MnmC(o)-like enzymes in those organisms lacking *mnmC(o)* and map their phylogenetic distribution.

### Substrate specificity of MnmM

Based on our ASL-bound MnmM structures, the enzyme appears to recognize the overall shape of the single-stranded anticodon loop of tRNA by forming multiple polar/ionic interactions with the backbone phosphate and ribose moieties along A31 through G36. Additionally, only a set of specific bases of ASL, namely U33, U34, and U35, are engaged in multiple intermolecular interactions with the protein. It has been shown that mnm^5^s^2^U is present in tRNA^Gln^ (UUG), tRNA^Glu^ (UUC), and tRNA^Lys^ (UUU) of *B. subtilis* or *S. aureus*. Interestingly, sequence analysis of tRNA genes in these organisms denotes that U33–U34–U35 are included in tRNA^Gln^ (UUG), tRNA^Glu^ (UUC), and tRNA^Lys^ (UUU) only. Therefore, our structural and biochemical data provide a plausible explanation in regard to the molecular basis for the specificity of MnmM. Meanwhile, *E. coli* MnmC displays a slightly different specificity; tRNA^Arg^ (UCU), tRNA^Glu^ (UUC), and tRNA^Lys^ (UUU) are substrates for both MnmC(o) and MnmC(m), whereas the major modification in tRNA^Gln^ (UUG) is cmnm^5^s^2^U as this isoacceptor is not a substrate for MnmC(o). It is challenging to decipher fundamental principles underlying the different specificity of MnmC(m) by extrapolating our findings with MnmM because of poor sequence homology and structural diversity (Cα RMSD of 5.06 Å). It would be imperative to obtain the tRNA-bound structure of MnmC to understand the structure-based mechanism of the distinct substrate selection.

### A proposed catalytic mechanism of MnmM

**Figure 7.**
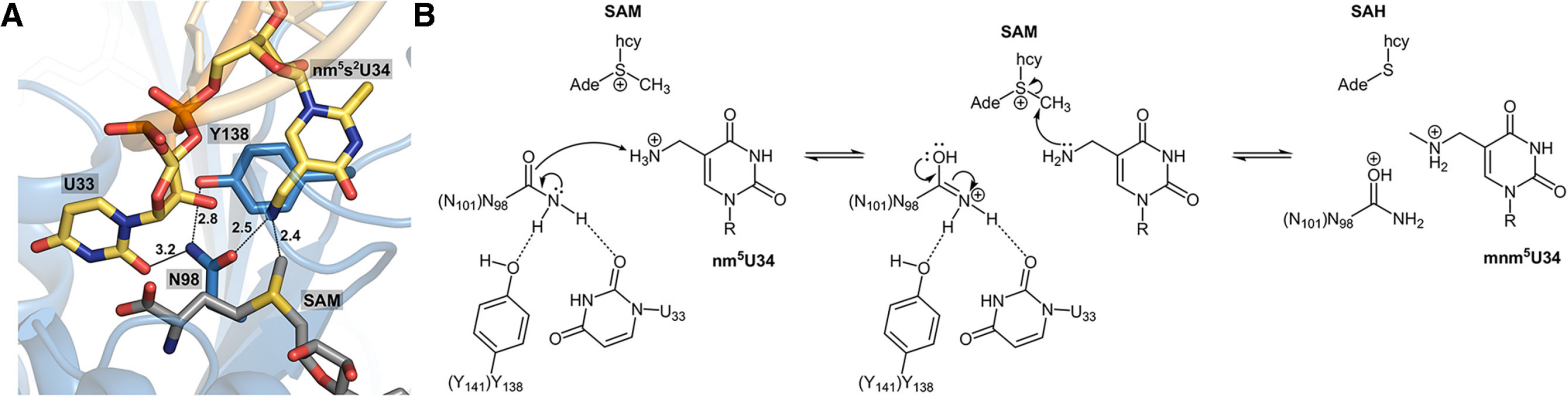
A proposed reaction mechanism for the methylation of *bs*MnmM. (**A**) A close-up of the active site of *bs*MnmM using a model with nm^5^s^2^U34 bearing tRNA. Hydrogen bonds are shown as dashed lines and the distances are labeled in Å. (**B**) In the first step, the oxyanionic form of the amide side chain of N98 (N101) deprotonates from the methyl ammonium group of nm^5^s^2^U34. Next, the activated amine in nm^5^s^2^U34 attacks the *S*-methyl group of SAM to complete the formation of mnm^5^s^2^U34 and SAH. The dotted lines represent hydrogen bonds. Ade, adenosine; hcy, homocysteine.

Our ASL-bound and ASL-free structures of MnmM disclose that the binding of cognate tRNA induces conformational changes in both the enzyme and the substrate. In particular, the β4–α5 and β5–α6 loops of MnmM become structurally organized and the bases of U33–U34 within the anticodon loop flip out from the stacked position. It is reasonable to consider that the ternary complex of MnmM-SAM-ASL mimics a Michaelis complex, except that it cannot proceed to the chemical step due to the lack of 5-aminomethyl modification. The presence of the 5-aminomethyl group on U34 is obviously essential for MnmM reaction, which is likely to require proper interactions with the enzyme for the productive binding and activation before the nucleophilic attack on the *S*-methyl group of SAM. For example, the deprotonation step of the 5-aminomethyl group must be critical, considering a rather high *pKa* value of the 5-aminomethyl group, which is estimated to be over 9 based on the values of 9.10 for cmnm^5^s^2^U and 9.51 for mnm^5^s^2^U (74). Notably, the negative electrostatic potential on the small pocket of the enzyme that accommodates the U34 base suggests the importance of facilitating the binding of the positively charged hypomodified uracil (Figure 4C–F). Since our co-crystal structures of MnmM with ASL were obtained from unmodified RNA, we attempted to build a model with tRNA bearing nm^5^s^2^U at the wobble position for better understanding of the enzyme mechanism (Figure 7A and Supplementary Figure S9). The model provides crucial clues on the identity of the general base, which can promote the nucleophilicity of nm^5^s^2^U. The carboxyl group of SAM or the nearest phosphate of tRNA is too distant from the amine of nm^5^s^2^U (6.8 and 4.4 Å, respectively), thus unlikely to be involved in the deprotonation step. Notably, the 2′-hydroxyl group of U33 is 2.9 Å from the substrate amine in our model. However, the hydroxyl group does not appear to be in an anionic environment, which must be necessary to increase its basicity. Meanwhile, the oxygen atom of the amide side chain of Asn-98 of *sa*MnmM (or Asn-101 of *bs*MnmM) is 2.5 Å away from the 5-aminomethyl group, which appears to be a key amino acid residue as supported by the mutagenesis experiments. Since no other residue is available in the vicinity, we speculate that the oxyanion form of the amide side chain may act as a general base for the nm^5^s^2^U activation (Figure 7B). There have been a few reports on the asparagine residue acting as a general base such as 1,2-α-l-fucosidase, where the intrinsic resonance for the amide side chain of the asparagine residue was demonstrated to be the source of the catalytic activity through quantum mechanical and molecular mechanical (QM/MM) simulation (75). Another example features a glycoside hydrolase PcCel45A, where the basicity of the asparagine residue via amide tautomerization was supported by high-resolution X-ray and neutron crystal structures (76) and QM/MM method (77). The basicity of the asparagine side chain of MnmM may be further enhanced by hydrogen bond acceptors like Tyr-141 (or Tyr-138 in *bs*MnmM) and U33 as shown in our structures. The necessity of the tyrosine residue was further demonstrated by mutagenesis experiments, where the 4-hydroxyl group in the phenol side chain is essential for the activity. Likewise, our proposed catalytic mechanism underscores the importance of U33 of tRNA in the chemical step, providing an additional reason why it is a part of the specificity element for MnmM reaction.

## DATA AVAILABILITY

The atomic coordinates and structure factors for *bs*MnmM-SAH, *bs*MnmM-SAM-ASL, *sa*MnmM-apo, *sa*MnmM-SAM, and *sa*MnmM-SAM-ASL are deposited to Protein Data Bank (PDB) under accession code 8H0T, 8H0S, 8H1A, 8H27, and 8H1B, respectively.

## Supplementary Material

gkad048_Supplemental_Files
