# Supplementary material for: Identification of a novel 5-aminomethyl-2-thiouridine methyltransferase in tRNA modification
Source: Nucleic Acids Res. 2023 Feb 10;51(4):1971–83. doi: 10.1093/nar/gkad048 (PMC9976899; doi:10.1093/nar/gkad048)
Supplement: gkad048_Supplemental_Files [file gkad048_supplemental_files.zip › Supplementary figures.docx]

**Supplementary Figures**

**Identification of a novel 5-aminomethyl-2-thiouridine methyltransferase in tRNA modification**

Gyuhyeok Cho^1†^, Jangmin Lee^1†^, and Jungwook Kim^1*^

^1^ Department of Chemistry, Gwangju Institute of Science and Technology, Gwangju, 61005, Korea.

^†^ These authors contributed equally to this work

* To whom correspondence should be addressed. Tel: +82-062-715-4622; Fax: +82-062-715-2866; Email: jwkim@gist.ac.kr


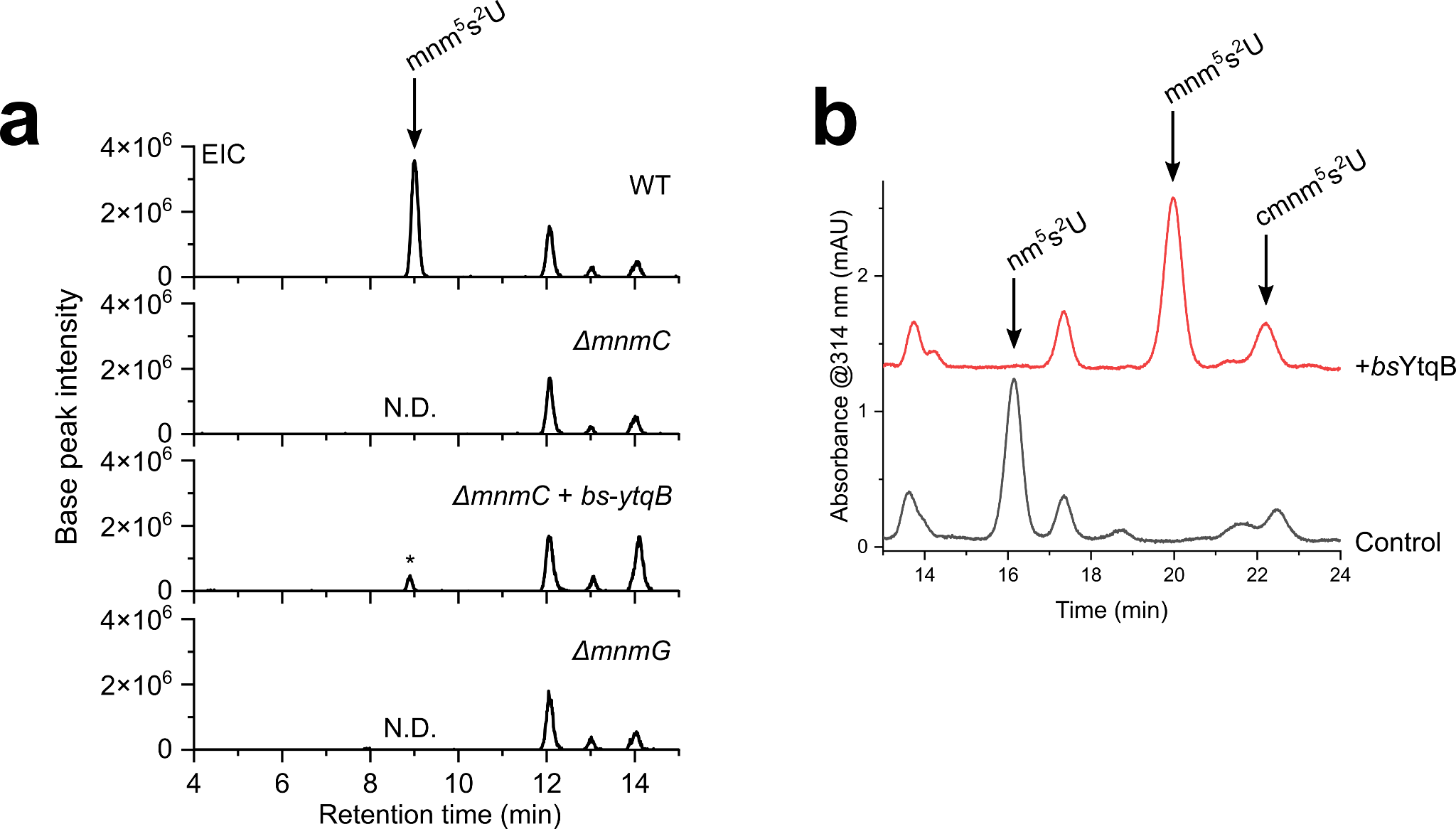


**Supplementary figure S1.** LC-MS and HPLC analysis of *E. coli* gene complementation and in vitro methylation assays of *B. subtilis* YtqB. Related to Figure 3.

**a.** Extracted ion chromatography (EIC) of mnm^5^s^2^U (m/z 304.09617±0.01) were extracted from LC-MS analysis of bulk tRNA hydrolysates which obtained from *E. coli* wild-type, *ΔmnmC*, *ΔmnmC* transformed with a plasmid pQE-*bs*-*ytqB*, and *ΔmnmG* strains. The asterisk designates mnm^5^s^2^U. N.D., not detected. **b.** HPLC analysis of in vitro methylation experiments of *bs*YtqB using bulk tRNA extracted from *E. coli* *ΔmnmC* as a substrate.


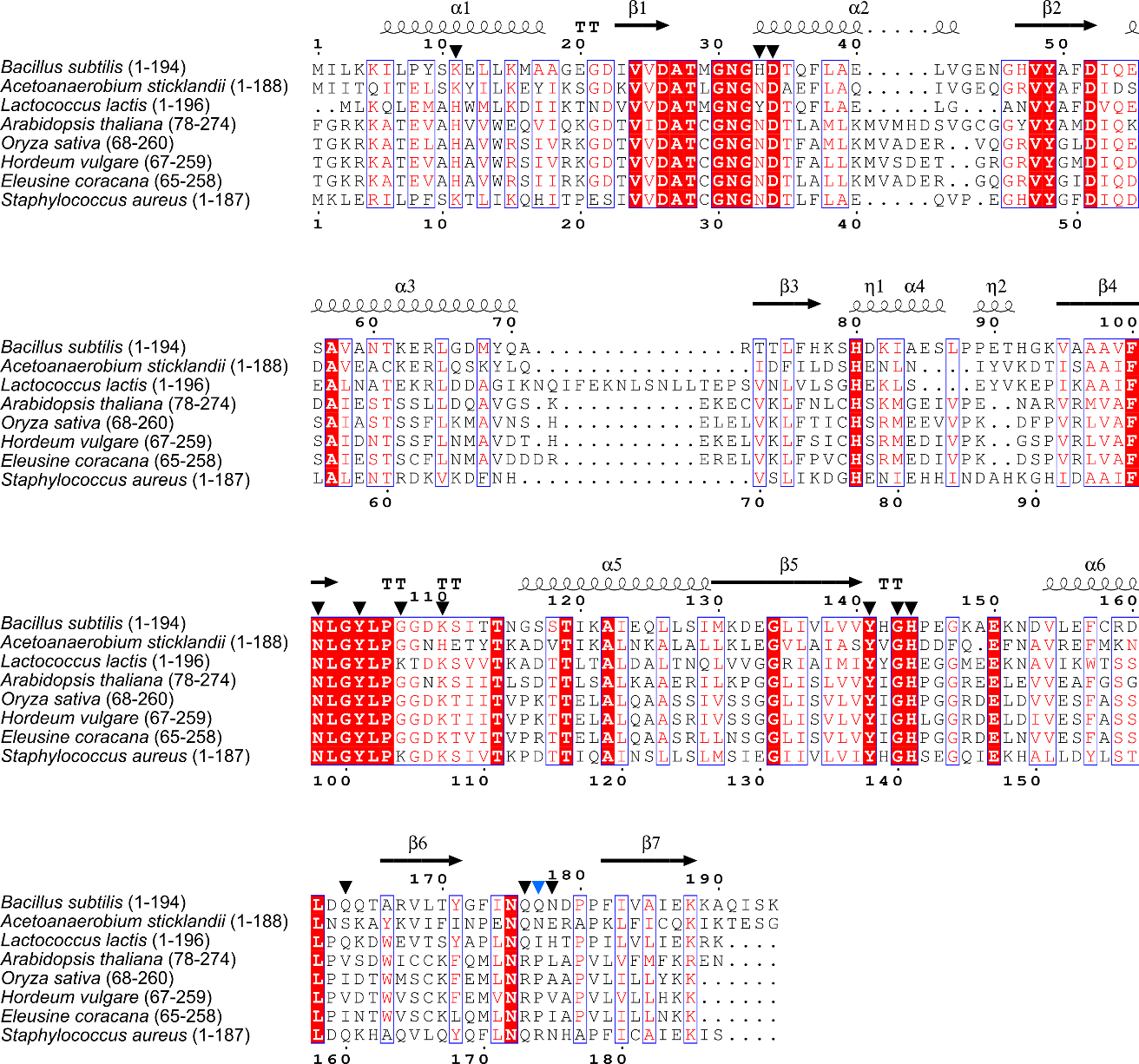


**Supplementary figure S2. Multiple sequence alignments of consensus sequences of MnmMs**

Consensus protein sequences from homologs of *bs*MnmM were aligned by Clustal Omega and visualized by ESPript3.0 (1). Residue numbers of bsMnmM or saMnmM were labeled on the top or the bottom of the aligned sequences, respectively. The secondary structure of bsMnmM was labeled over the aligned sequences. The residues interacting with ASL were marked in black or blue triangles observed in both of the complex structures or only in the bsMnmM-SAM-ASL, respectively. UniProt or NCBI sequence IDs that aligned are O34614 (*Bacillus subtilis*), Q2FXG9 (*Staphylococcus aureus*), E3PX47 (*Acetoanaerobium sticklandii*), A0A0A7T323 (*Lactococcus lactis*), Q8GUP2 (*Arabidopsis thaliana*), Q0DGU2 (*Oryza sativa*), F2EG75 (*Hordeum vulgare*), and GJM89025.1 (*Eleusine coracana*). Ranges of amino acid residues used for the alignments are numbered in parenthesis.


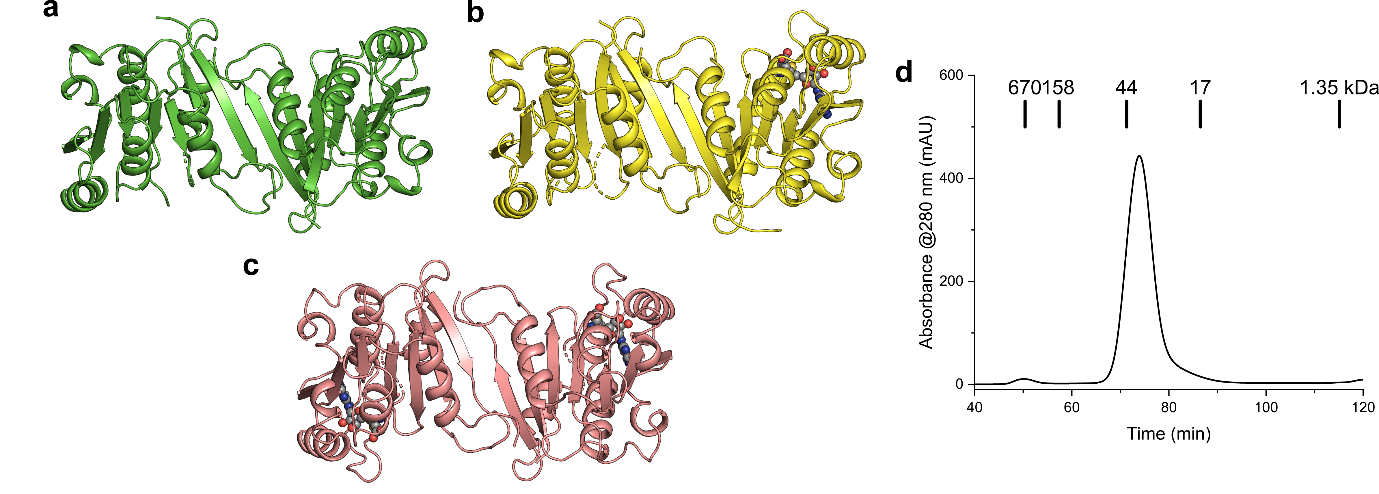


**Supplementary figure S3.** Overall structures of **a.** *sa*MnmM Apo (green) **b.** *sa*MnmM-SAM (yellow) and **c.** *bs*MnmM-SAH (salmon). Proteins are displayed in cartoon and bound ligands in sphere.

**d.** Gel filtration chromatography graph of *sa*MnmM. Oligomeric state of *sa*MnmM in solution imply as dimer. Molecular weight of *sa*MnmM is 21.9 kDa.


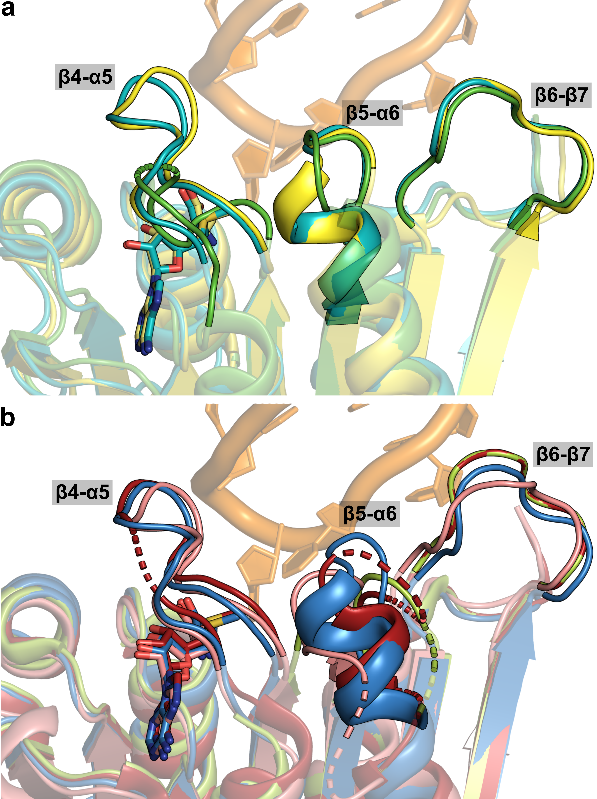


**Supplementary figure S4. Comparison of SAM and ASL binding site, β4-α5 and β5-α6 loops, in various structures.**

**a.** Cartoon image overlay of saMnmM structures. *sa*MnmM-SAM-ASL (cyan), *sa*MnmM-Apo (green), *sa*MnmM-SAM (yellow). Structural comparison reveals that SAM or SAH binding induce relatively rigid structures in β4-α5 and β5-α6 loops.

**b.** Cartoon image overlay of *bs*MnmM structures. *bs*MnmM-SAM-ASL (deep blue), 4PON (lime), 4POO (red), *bs*MnmM-SAH (salmon).


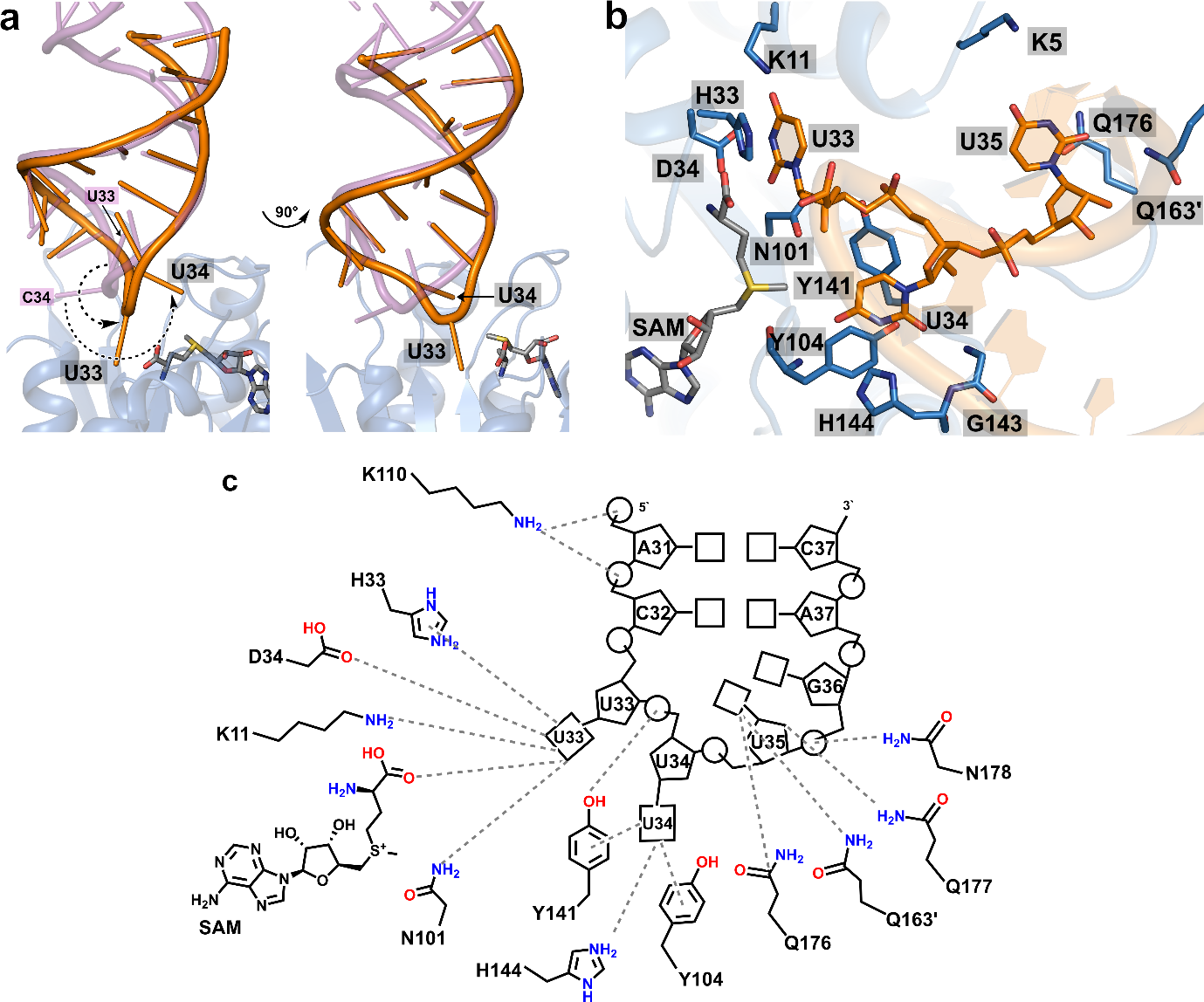


**Supplementary figure S5. Base flipping of U33 and U34 in anticodon loop upon binding to *bs*MnmM** a. Active site of *bs*MnmM-SAM-ASL highlighting the interactions with U33, U34, and U35 of bound ASL (carbon atoms in orange) and SAM (carbon atoms in grey), compared with ribosome binding tRNA (4V7M, purple). Bases of both U33 and U34 are flipped-out in the ASL-*bs*MnmM complex structure. b. Close-up of the active site showing the molecular interactions among the anticodon loop of tRNA, *bs*MnmM, and SAM. c. Schematic diagram of interactions between the ASL with amino acid residues of *bs*MnmM. Oxygen is shown in red, nitrogen in blue, and sulfur in yellow.


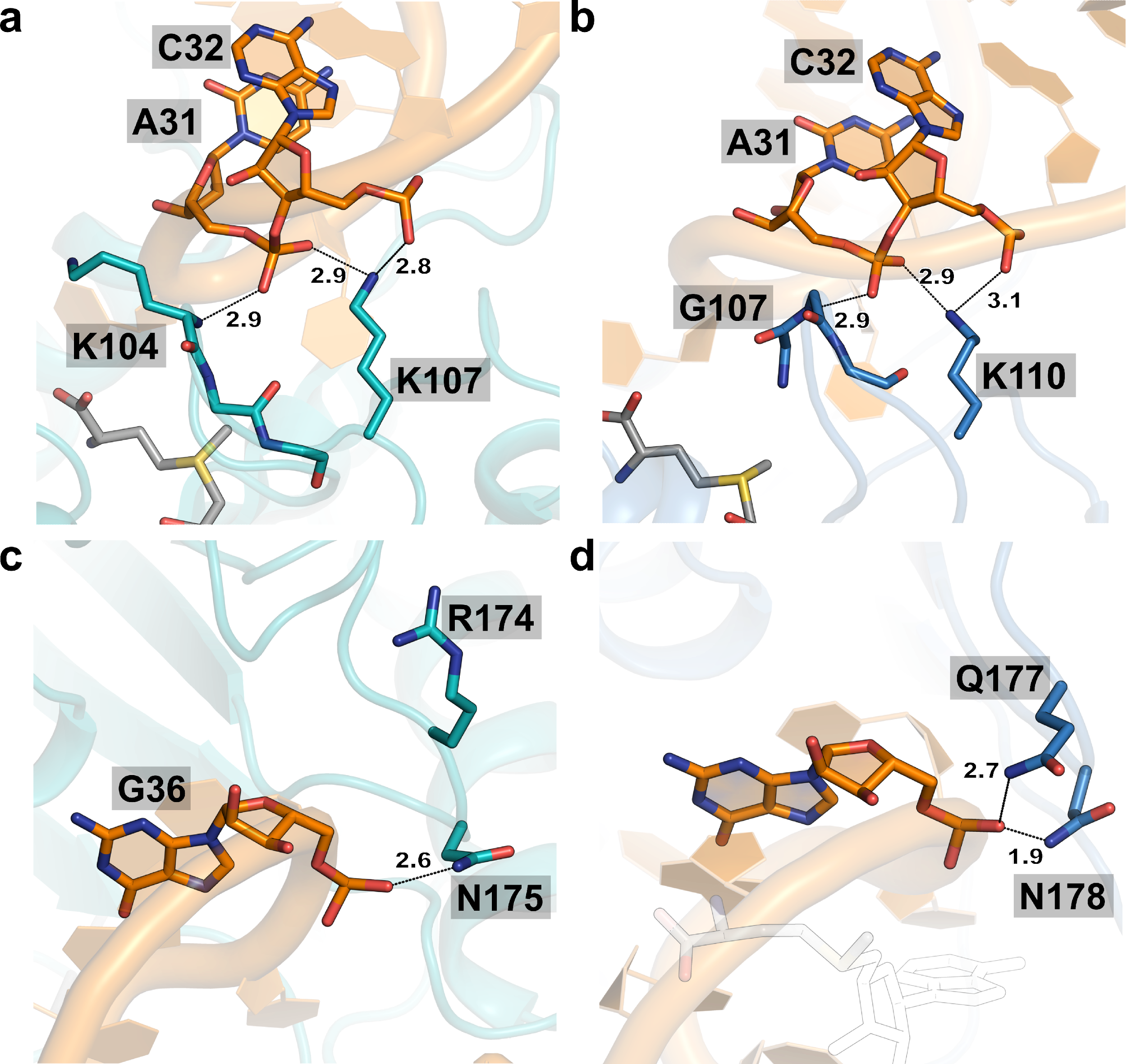


**Supplementary figure S6. Interactions of MnmM with nucleotides 31, 32, 35, and 36 of ASL**

Interactions between the **a.** K107 of *sa*MnmM and **b.** K110 of *bs*MnmM with A31 and C32 nucleotides. U35 and G36 interactions with **c.** *sa*MnmM and **d.** *bs*MnmM residues. Hydrogen bonds are shown as gray dashed lines.


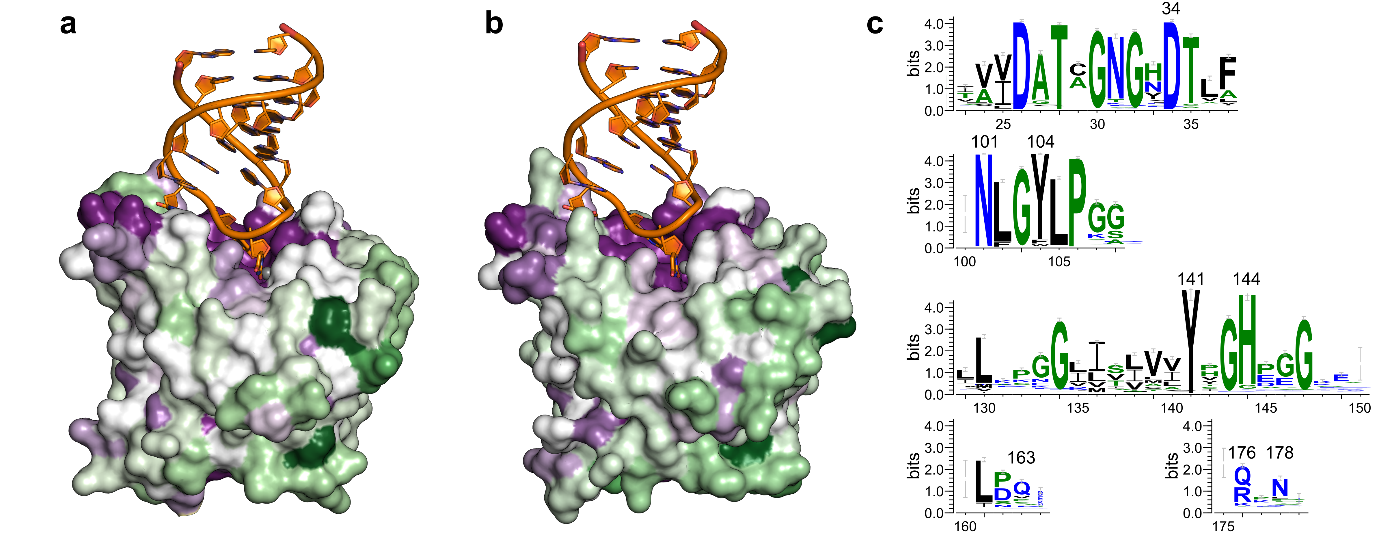


**Supplementary figure S7. Conservation amino acid analysis of MnmMs**

Conservation scores of amino acid sequences were calculated by ConSurf (2) and mapped on the surface of the **a.** *sa*MnmM-SAM-ASL or **b.** *bs*MnmM-SAM-ASL structures. **c.** Highly conserved residues of MnmM (rRNA methylase; InterPro ID: IPR010719) were plotted by WebLogo3 (3). Total 1640 sequences from NCBI RefSeq genomes (4) were used for alignments. Residues are numbered in *bs*MnmM.


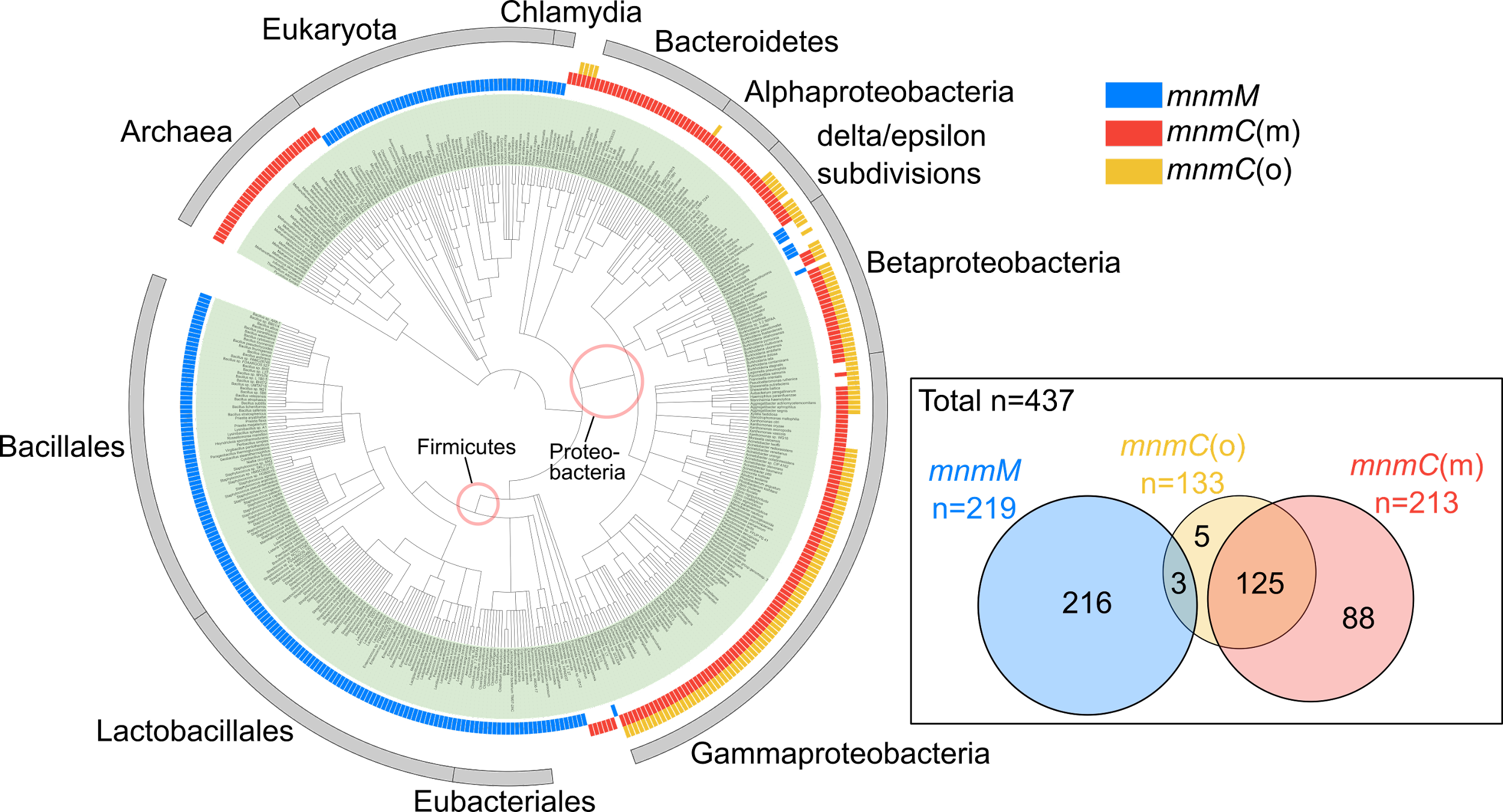


**Supplementary figure S8. Phylogenetic distribution of *mnmM*, *mnmC*(SAM), and *mnmC(*FAD)**

(a) Gene occurrence profiles are plotted to phylogenetic tree of 437 species from the three domains of life. Proteins in NCBI RefSeq genomes (4) containing corresponding domains were obtained from UniProt (5); *mnmM*, Putative rRNA methylase (InterPro ID: IPR010719); *mnmC*(SAM), MnmC-like methyltransferase (IPR008471); *mnmC*(FAD), tRNA U-34 5-methylaminomethyl-2-thiouridine biosynthesis protein MnmC, C-terminal (IPR017610). Taxallnomy (6) were used to retrieve a list of species from taxonomic ID of the obtained protein entries. Based on the retrieved species, a phylogenetic tree was built by NCBI Common tree tool. iTOL (7) was used to visualize the tree and to plot the gene occurrence profiles. Presence of *mnmM*, *mnmC(*SAM), and *mnmC*(FAD) was labeled as color strips in blue, red, and orange, respectively. Representative taxonomic ranks are labeled as gray strip. Venn diagram of the species containing the number of each domain is shown in the right rectangle. *mnmM* and *mnmC*(SAM) are exclusively distributed in the listed species. In 3 species from Neisseriaceae, *mnmM* and *mnmC*(FAD) co-occurred. The organisms and gene occurrence profiles are listed in **Supplementary table S6**.


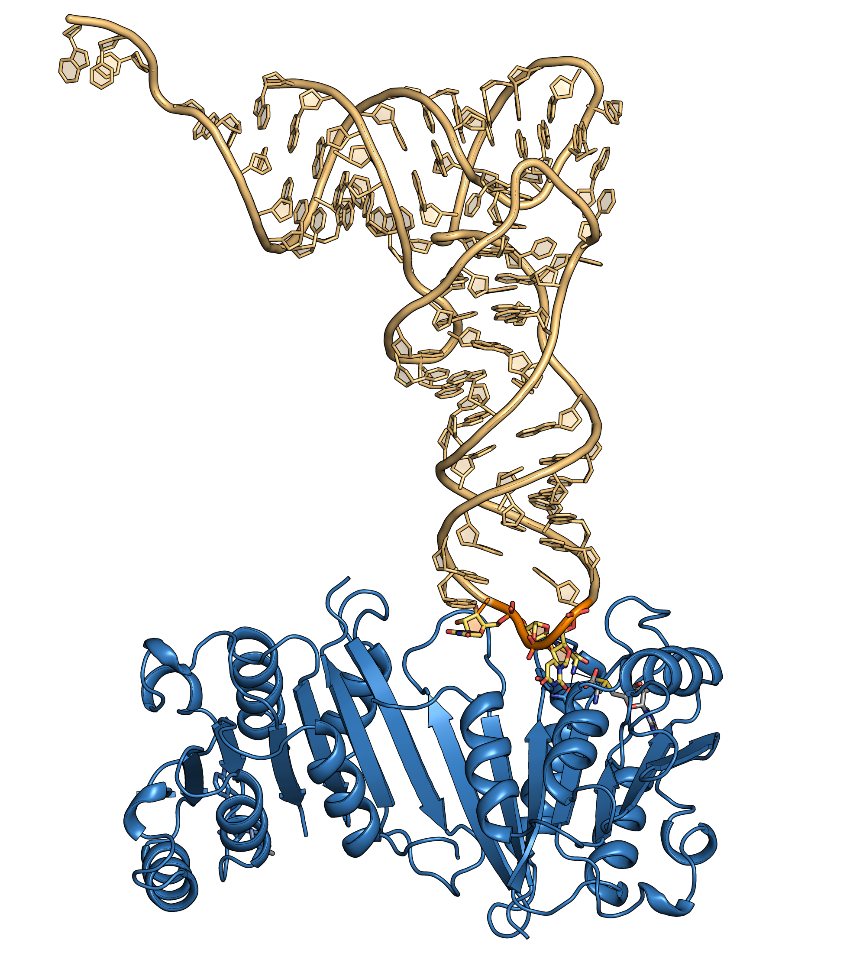


**Supplementary figure S9. nm^5^s^2^U34-tRNA interaction model with *bs*MnmM complex structure. Related to Figure 7A.**

A model for full-length tRNA binding to *bs*MnmM. ASL region of ribosome-bound tRNA (PDB: 4V7M, Chain ID: AW) were aligned to *bs*MnmM-SAM-ASL using Coot (8). nm^5^s^2^U modification was introduced by deleting terminal 5-methyl group of mnm^5^s^2^U (Ligand ID: U8U).

**Supplementary Figures References**

1. Robert,X. and Gouet,P. (2014) Deciphering key features in protein structures with the new ENDscript server. *Nucleic Acids Res.*, **42**, W320–W324.

2. Ashkenazy,H., Abadi,S., Martz,E., Chay,O., Mayrose,I., Pupko,T. and Ben-Tal,N. (2016) ConSurf 2016: an improved methodology to estimate and visualize evolutionary conservation in macromolecules. *Nucleic Acids Res.*, **44**, W344–W350.

3. Crooks,G.E., Hon,G., Chandonia,J.M. and Brenner,S.E. (2004) WebLogo: A sequence logo generator. *Genome Res.*, **14**, 1188–1190.

4. Maglott,D., Ostell,J., Pruitt,K.D. and Tatusova,T. (2011) Entrez gene: Gene-centered information at NCBI. *Nucleic Acids Res.*, **39**, D52–D57.

5. Bateman,A., Martin,M.J., Orchard,S., Magrane,M., Agivetova,R., Ahmad,S., Alpi,E., Bowler-Barnett,E.H., Britto,R., Bursteinas,B., *et al.* (2021) UniProt: the universal protein knowledgebase in 2021. *Nucleic Acids Res.*, **49**, D480–D489.

6. Sakamoto,T. and Ortega,J.M. (2021) Taxallnomy: an extension of NCBI Taxonomy that produces a hierarchically complete taxonomic tree. *BMC Bioinformatics*, **22**, 1–23.

7. Letunic,I. and Bork,P. (2021) Interactive tree of life (iTOL) v5: An online tool for phylogenetic tree display and annotation. *Nucleic Acids Res.*, **49**, W293–W296.

8. Emsley,P., Lohkamp,B., Scott,W.G. and Cowtan,K. (2010) Features and development of Coot. *Acta Crystallogr. Sect. D Biol. Crystallogr.*, **66**, 486–501.
